# COX-2 induces oncogenic micro RNA miR655 in human breast cancer

**DOI:** 10.1038/s41598-017-18612-3

**Published:** 2018-01-10

**Authors:** Mousumi Majumder, Leanna Dunn, Ling Liu, Asma Hasan, Krista Vincent, Muriel Brackstone, David Hess, Peeyush K. Lala

**Affiliations:** 1 Department of Biology, Brandon University, Brandon, Manitoba Canada; 2 Department of Anatomy and Cell Biology, Schulich School of Medicine and Dentistry, University of Western Ontario, London, Ontario Canada; 3 Department of Oncology, University of Alberta, Edmonton, Alberta Canada; 4 Department of Oncology, Schulich School of Medicine and Dentistry, University of Western Ontario, London, Ontario Canada; 5 Lawson Research Institute, London, Ontario Canada; 6 Physiology and Pharmacology, Schulich School of Medicine and Dentistry, University of Western Ontario, London, Ontario Canada; 7 Robarts Research Institute, London, Ontario Canada

## Abstract

We show that Cyclooxygenase-2 over-expression induces an oncogenic microRNA miR655 in human breast cancer cells by activation of EP4. MiR655 expression positively correlated with COX-2 in genetically disparate breast cancer cell lines and increased in all cell lines when grown as spheroids, implicating its link with stem-like cells (SLCs). Ectopic miR655 over-expression in MCF7 and SKBR3 cells resulted in increased proliferation, migration, invasion, spheroid formation and Epithelial to Masenchymal transition (EMT). Conversely, knocking down miR655 in aggressive MCF7-COX2 and SKBR3-COX2 cells reverted these phenotypes. MCF7-miR655 cells displayed upregulated NOTCH/WNT genes; both pathway inhibitors abrogated miR655-induced spheroid formation, linking miR655 with SLC-related pathways. MiR655 expression was dependent on EP4 activity and EP4 downstream signaling pathways PI3K/AKT, ERK and NF-kB and led to TGFβ resistance for Smad3 phosphorylation. Tail vein injection of MCF7-miR655 and SKBR3-miR655 cells in NOD/SCID/GUSB-null mice revealed increased lung colony growth and micrometastases to liver and spleen. MiR655 expression was significantly high in human breast tumors (n = 105) compared to non-tumor tissues (n = 20) and associated with reduced patient survival. Thus miR655 could serve as a prognostic breast cancer biomarker.

## Introduction

Breast cancer accounts for the second highest cause of cancer-related mortality in women in North America^1^. Cyclo-oxygenase (COX)-2, an inflammation-inducible enzyme, is upregulated in approximately 40% of breast cancer^2,3^ including ductal carcinoma *in situ* ^3,4^ and invasive carcinomas^5^, correlated with disease progression and metastasis^6^ and reduced patient survival^2,3^. While intake of COX-2 inhibitors was shown to have chemo-protective effects against breast cancer development and morbidity^7^, they can cause thrombo-embolic side effects^8,9^, suggesting the need of identifying better alternative target(s) downstream of COX-2. Prostaglandin E (PGE)2, the chief prostanoid resulting from COX-2 activity binds to four G-protein coupled PGE receptors, EP1-4^10–12^, having differential signaling abilities. EP1 couples with Gq, activating PLC and a rise in intracellular Ca^2+^, whereas EP2 and EP4 couple with Gs, stimulating cAMP/PKA pathway and most EP3 isoforms couple with Gi, inhibiting cAMP. Additionally, EP4 also stimulates non-canonical pathways PI3K/Akt and ERK^12^, promoting cell survival and migration^13,14^. We found that EP4 is an ideal therapeutic target to replace COX-2 for three reasons: (1) it accounts for most of the COX-2 mediated events in breast cancer progression; (2) it is relatively redundant for most physiological functions of COX-2 shared by EP2 via cAMP/PKA pathway; (3) it does not bind to cardio-protective prostanoid PGI2^15^, depleted by COX-2 inhibitors. PGE2 mediated EP4 activation on cancer or host cells resulting from aberrant COX-2 activity was shown to promote multiple cellular events responsible for breast cancer progression: paralysis of host anti-tumor immune cells^16,17^, stimulation of tumor cell migration and invasiveness^18,19^, tumor-associated angiogenesis and lymphangiogenesis resulting from upregulation of lymphangiogenic factors VEGF-C or VEGF-D in cancer cells^20–22^ or tumor-infiltrating macrophages^22^, and finally, an induction of stem-like cells (SLC) phenotype^23,24^. We showed that EP4 antagonists at nontoxic doses exerted strong anti-tumor, anti-metastatic and SLC-reductive effects in a COX-2 expressing, highly metastatic, syngeneic murine breast cancer model^22^.

MicroRNAs (miRNAs; miRs) are short, non-coding regulatory RNAs that down-regulate gene expression at the post-transcriptional level, some emerging as potential biomarkers of cancer^25^. Genes coding for miRNAs are frequently located in fragile sites of chromosomes, having increased susceptibility to mutation^26^ making them the key candidates for carcinogenesis. In human breast cancer, miRNA expression profiles were reported to be distinct for basal and luminal sub-types, ER and HER2 status, and predict responses to chemotherapies^27,28^. Altered miRNA expression can influence several steps of the metastatic cascade, including cell adhesion, EMT, motility, invasiveness, and resistance to apoptosis^29–33^. Some of them were shown to play an important regulatory role on EMT phenotype in human breast^33^ and colon cancer^34^. MiRNAs can appear in circulation via exosomes, some reported to mark early stage breast cancer^35,36^.

By differential gene and miRNA microarray analyses of MCF7 and ectopic COX-2 over-expressing MCF-7-COX-2 cells we identified two COX-2 upregulated miRNAs, miR526b and miR665, of which miR526b was established as an oncogenic miRNA in human breast cancer^37^. Stable overexpression of miR526b in poorly metastatic, low miRNA expressing cell lines resulted in increased cellular migration, invasion, EMT phenotype and enhanced tumorsphere formation *in vitro*, and lung colony formation *in vivo* in immunodeficient mice. Conversely, knockdown of miR526b in highly aggressive COX-2/miRNA over-expressing cells reduced oncogenic functions and reversed the EMT phenotype. MiR526b expression was dependent on EP4 receptor activity and downstream PKA, PI3K/Akt, and NF-κB pathways. Finally, miR526b expression was significantly higher in cancerous than in non-cancerous breast tissues and associated with reduced patient survival^37^.

Stem-like cells (SLCs) comprise a small subset of cells within the tumor, believed to be capable of unlimited self-renewal, to resist chemo- and radiation therapies that reduce tumor bulk by killing non-stem proliferating cells^38,39^. We believe that SLCs represent a dynamic cell population regulated by many molecules in the tumor microenvironment. We have shown that COX-2 or EP4 activity in breast cancer induces and sustains SLCs by activation of PI3K/Akt followed by NOTCH/WNT signaling pathways^23^. Certain miRNAs such as the Let7 family, and miR-200C were shown to be inversely associated with maintenance of SLCs in human breast cancer^40,41^. On the other hand, we found that COX2/EP4 induced oncogenic miR526b is also SLC-promoting in human breast cancer cells^37^. These findings reveal that certain miRNAs may serve as SLC-linked biomarkers in breast cancer.

Here we report the functions of miR655 as another oncogenic and SLC-promoting miRNA, which was significantly upregulated in COX-2-high human breast cancer cell lines, during natural as well as ectopic COX-2 over-expression. Both miR526b and miR655 are members of same miRNA cluster. The genes coding for both miRNAs are located on chromosome 19. In our preliminary findings conducted *in vitro* with human breast cancer cell lines^42^, miR655 was shown to have oncogenic and SLC-inducing properties. Contrary to our findings and data presented later in this article, miR655 was reported as an EMT suppressor in pancreatic cell lines^43^ by targeting Zeb-1 and an inhibitor of cellular invasion in squamous cell carcinoma cell lines by targeting pituitary tumor-transforming gene-1 (PTTG1)^44^. In a recent study in human breast cancer cell lines, this miRNA was reported to have an EMT suppressor role^45^. Here we present a detailed study of the functions of miR655 in human breast cancer employing miRNA-manipulated breast cancer cell lines tested *in vitro* and *in vivo* for changes in a variety of functions related to their oncogenic phenotypes. We also examined the relationship of miRNA expression in human breast cancer tissues with tumor grade and patient survival. Our results unequivocally demonstrate that miR655 is a COX-2-induced oncogenic miRNA linked with SLC-phenotype, up-regulated by EP4-mediated signaling pathway PI3K/AkT/NFκB and SLC pathway NOTCH/WNT upregulation and resulting in TGFβ resistance for Smad3 activation. MiR655 expression was elevated in primary breast cancer tissues, high expression being associated with reduced survival.

## Results

### Identification of miR655 upregulation in MCF7-COX-2 cells

Using miRNA micro array and gene expression arrays to compare ectopic COX-2 expressing MCF7-COX-2 and MCF7-Mock (empty plasmid expressing control) cells, we identified several miRNAs and genes whose expressions were differentially regulated, showing ±1.5-fold changes with nominal alpha value 0.05. We identified two miRNAs, miR526b and miR655 which were up-regulated in MCF7-COX-2 cells, along with several genes which were up- or down-regulated in the same cell line^23^. Genes targeted by miR655 are listed in Supplementary Table 1.

### Positive association of miR655 with COX-2 expression in multiple COX-2 disparate human breast cancer cell lines

We tested numerous COX-2 disparate human breast cancer cell lines varying in gene expression profile^46^ to explore whether miR655 expression levels were broadly correlated with COX-2 expression. Data presented in Supplementary Figure 1A reveal that this was indeed the case, suggesting that, amongst many genes, COX-2 may play an important role in miR655 up-regulation. That COX-2 activity was instrumental in this upregulation is shown later. We selected MCF7 (non-metastatic, low COX-2, HER-2 negative, low miR655), and SKBR3 (poorly metastatic, COX-2 negative, HER-2 positive, low miR655) cell lines for miRNA over-expression.

### Validation of stable miR655 over-expression in MCF7 and SKBR3 cells

Stable over-expression of miR655 in MCF7 and SKBR3 was achieved using nucelofection^37^ and named as MCF7-miR655 and SKBR3-miR655. Empty vector transfected cells were respectively named as MCF7-Mock and SKBR3-Mock. Over-expression of miR655 was confirmed in both cell lines using real-time polymerase chain reaction (RT-PCR) in which RNU44 and RNU48 serving as control miRs (Supplementary Figure 1B).

### MiR655 overexpression promotes cellular migration, invasion and proliferation

**Figure 1:**
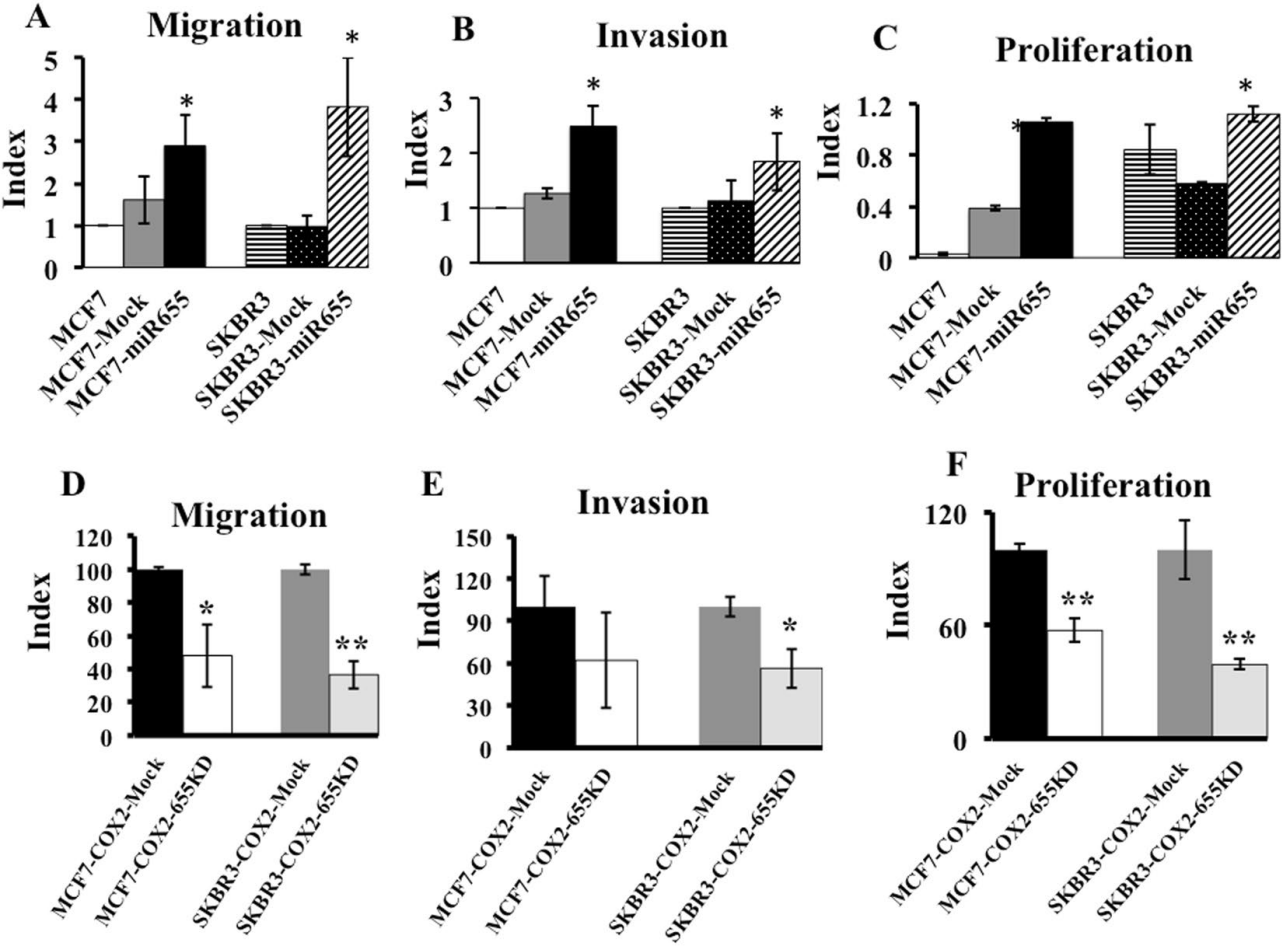
MiR655 over-expression promotes cellular migration, invasion and proliferation, and knock-down of miR655 in COX-2 high cells reduce aggressive phenotypes. Comparison of (**A**) migration, (**B**) invasion and (**C**) proliferation of parental, empty vector (Mock) transfected, and miR655 over-expressing MCF7 and SKBR3 cells. Comparison of (**D**) migration, (**E**) invasion and (**F**) proliferation of MCF7-COX-2-655KD and SKBR3-COX-2-655KD, compared to Mock cells. The data are presented as a mean of triplicate independent experiments ± SEM. *Indicates p < 0.05, **indicates p < 0.01 compared to parental and Mock cell lines.

To investigate an association between miR655 expressions with key steps in metastasis, transwell migration and invasion assays were performed, as reported previously^18,19,21–23^, with MCF7-miR655, SKBR3-miR655 and their respective Mock cells. Over expression of miR655 in both cell lines resulted in a significant increase in cellular migration (Fig. 1A), invasion (Fig. 1B and proliferation (Fig. 1C) measured with BrdU ELISA assay^23,47^.

### MiR655 knock-down in MCF7-COX-2 and SKBR3-COX-2 cells reduces cellular migration, invasion and proliferation

Morpholino-mediated knock-down of miR655 in high COX-2 expressing MCF7-COX-2 and SKBR3-COX-2 cell lines was confirmed using Taqman miRNA expression assays^37^. MiR655 was significantly downregulated in both cell lines at two different morpholino concentrations in a dose dependent way (Supplementary Fig. 1C), compared to mock transfected cells. We used 20 μM Morpholino transfected cells for further functional analysis. MiR655 downregulated cell lines were named as MCF7-COX-2-655KD, SKBR3-COX-2-655KD, and their respective empty vector controls named as MCF7-COX-2-Mock and SKBR3-COX-2-Mock. Knock-down of miR655 in both MCF7-COX-2 and SKBR3-COX-2 cells resulted in significantly reduced abilities for migration (Fig. 1D), invasion (Fig. 1E) and proliferation (Fig. 1F).

### Epithelial to Masenchymal Transition (EMT) in miR655 manipulated cells

**Figure 2:**
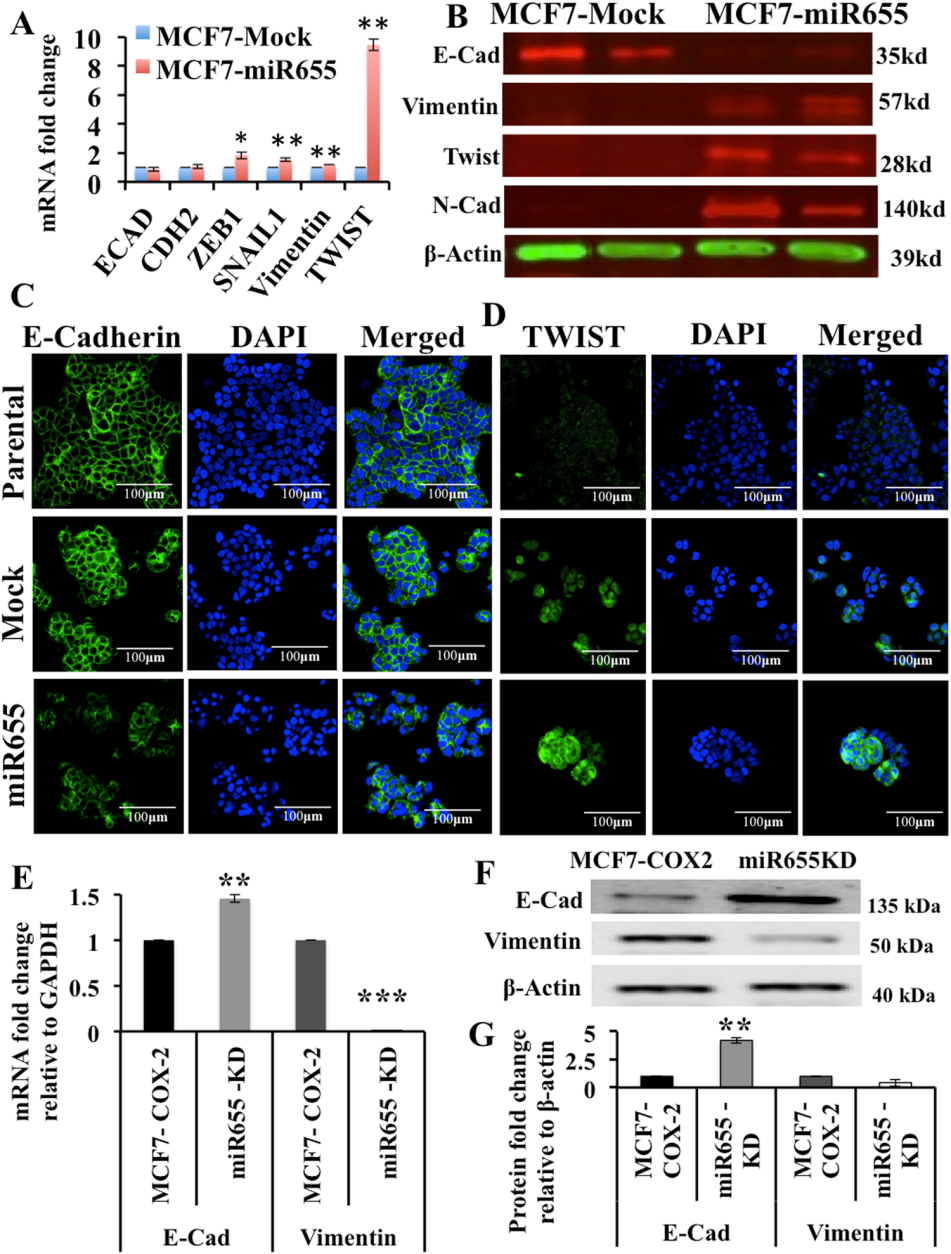
MiR655 over-expression promotes EMT in MCF7 cells and miR655 knock-down in MCF7-COX2 cells reverts to MET. Fold change in *E-Cadherin*, *N-Cadherin* (*CDH2*), *ZEB1*, *Vimentin* and *SNAIL* mRNA (**A**) and protein expression (except ZEB1 and SNAIL) (**B**) in MCF7-miR655 cells indicating EMT. Immunofluorescence assay showing down regulation of E-Cadherin (**C**) and upregulation of TWIST1 (**D**) in MCF7-miR655 cells. Knock down of miR655 in MCF7-COX2 cells up regulates *E-Cadherin* and down regulates *Vimentin* mRNA (**E**) and protein, measured with Western Blots (**F**), quantification presented in (**G**). For quantification, all comparisons were done relative to respective Mock cell lines. In figures (**B**) and (**F**), each marker was run on separate western blot and full western blots for Vimentin and E-Cadherin are shown in Supplementary Figures 3 & 4 respectively. Quantitative data are presented as a mean of triplicate independent experiments ± SEM. *Indicates p < 0.05, **p < 0.01 compared to parental and mock cell lines. Scale bar is 100 µm.

Although miR655 has no binding site for *E-Cadherin* (E-Cad) mRNA, it binds to the 3′UTR of *CTNNB1* catenin (cadherin-associated), beta 1. The protein encoded by this gene is part of a complex of proteins that constitute adherens junction in epithelial cells. It was also reported that miR655 targets and down regulates *ZEB1*, a negative regulator of E-Cad, resulting in upregulation of E-Cad in miR655 cells^43^. Hence, we decided to screen the well-known epithelial marker E-Cad and mesenchymal markers Vimentin, TWIST1, ZEB1 and SNAIL, N-Cadherin in miRNA-manipulated MCF7 cells. MiR655 over expression in MCF7 cells induced EMT, evidenced by decrease in E-Cad and increase in Vimentin, TWIST, ZEB1, SNAIL and N-Cadherin; mRNA data represented in Fig. 2A and protein in Fig. 2B,C. Since our ZEB1 and SNAIL antibody did not work, we have mRNA data only for these two molecules. Conversely, knockdown of miR655 in MCF7-COX-2 cells resulted in a significant increase in the E-Cad and decrease in Vimentin (Fig. 2E–G), indicating mesenchymal to epithelial transition (MET) or a reversal of their EMT phenotype. Together, these results demonstrate an important role for miR655 in promoting an aggressive phenotype in human breast cancer by inducing EMT.

### Link between miR655 and stem-like cell (SLC) phenotype

**Figure 3:**
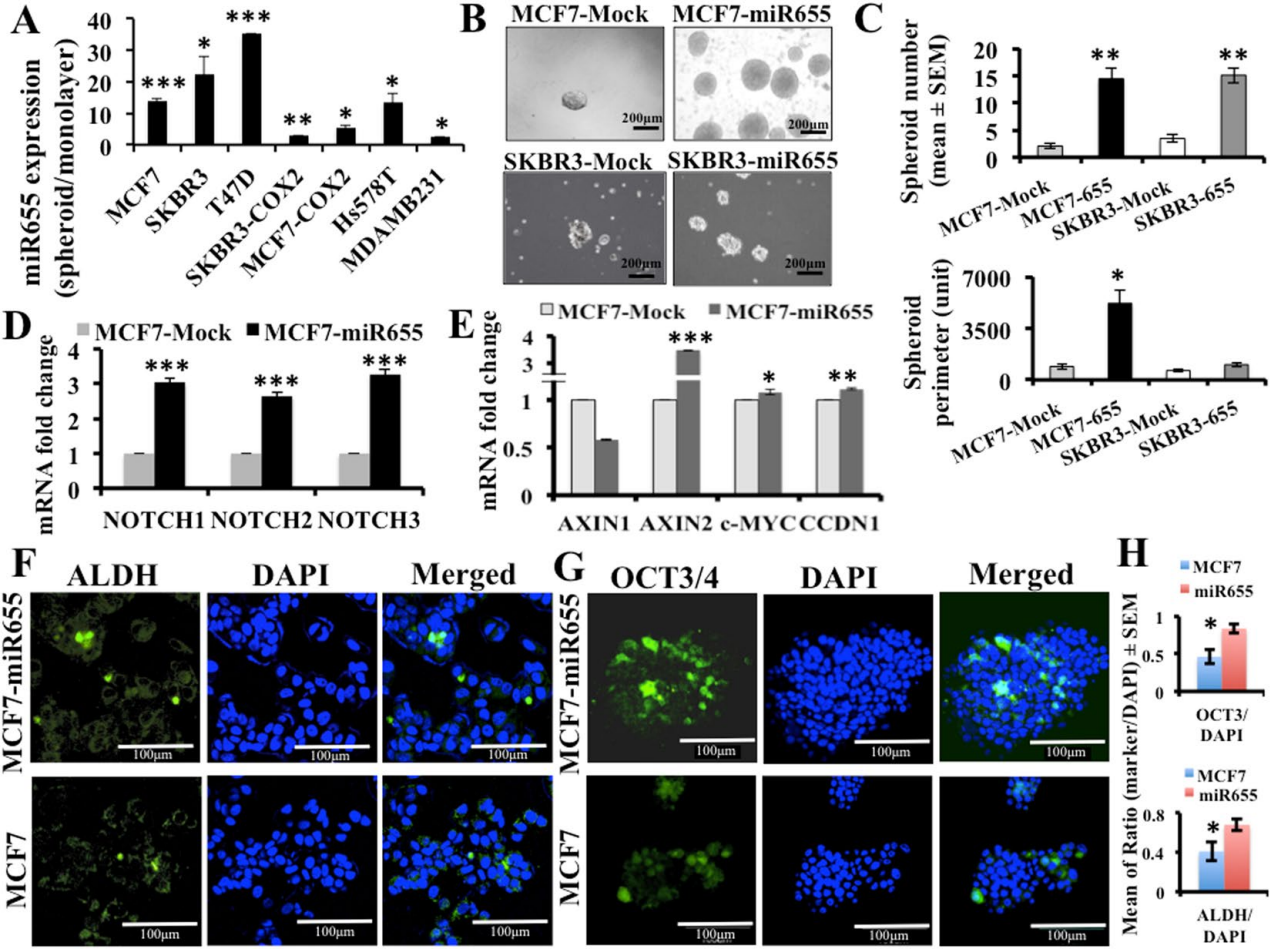
MiR655 over-expression induces stem-like cell phenotype in MCF7 and SKBR3 cells. (**A**) MiR655 expression tested in spheroids relative to monolayer culture conditions in breast cancer cell lines. (**B**) Comparison of spheroid formation by miR655 overexpressing cells and respective control cells presented as spheroid numbers (**C**) and sizes (perimeter). (**C**) Expression of NOTCH pathway genes (*NOTCH1, 2, 3*) and (**D**) WNT genes (*AXIN1, AXIN2, CCDN1* and *c-MYC*) in MCF7-miR655 cells. We identified SLC marker positive cells ALDH (**F**) and OCT3/4 (**G**) in MCF7 and MCF7-miR655 cells with immunofluorescence assay. Incidence of marker positive cells is presented in (**H**). Quantitative data are presented in as a mean of triplicate independent experiments ± SEM. *Indicates p < 0.05, **p < 0.005 and ***p < 0.001 compared to parental or mock or monolayer cell lines. Scale bar is 60 µm.

The closest *in vitro* assay of SLC function is the ability of single cells to form spheroid-like structures or tumorspheres or spheroids^48^. We allowed multiple human breast cancer cell lines to grow in regular monolayer culture condition and also in ultra-low attachment plates to grow as tumor spheroids, followed by miRNA quantification. In spheroids miR655 expression increased in all cell lines including COX-2-high and miRNA-high cells, however, this was more pronounced in COX-2-low and miRNA low cell lines (Fig. 3A). These results gave us the first clue of a positive association of SLC phenotype with miR655 upregulation in human breast cancer cells.

### MiR655 stimulates SLC phenotype in MCF7 and SKBR3 cells

To test the role of miR655 in SLC induction, MCF7-miR655 and SKBR3-miR655 human breast cancer cell lines, and their respective Mock and parental controls were plated in 6-well ultra-low attachment plates to perform spheroid formation assay. Compared to controls, both miR655 over-expressing cell lines displayed a significant increase in the number of spheroids (Fig. 3B,C), as well as spheroid sizes (given by average perimeter, Fig. 3C). We also observed an increase in NOTCH (Fig. 3D) and WNT genes (*c-MYC*, *CCDN1, AXIN2*), however *AXIN1* was downregulated in MCF7-miR655 cells (Fig. 3E). These two pathways were known to be associated with COX-2 induced SLC functions^21^. We also estimated the frequency of well-recognized breast cancer SLC marker ALDH^49^ and a pluripotency marker OCT3/4^50^ in MCF7 and MCF7-miR655 cell lines with immunostaining. A small population of ALDH (Fig. 3F) and OCT3/4 (Fig. 3G) cells were elevated in MCF7-miR655 cells compared to MCF7 (Fig. 3H).

**Figure 4:**
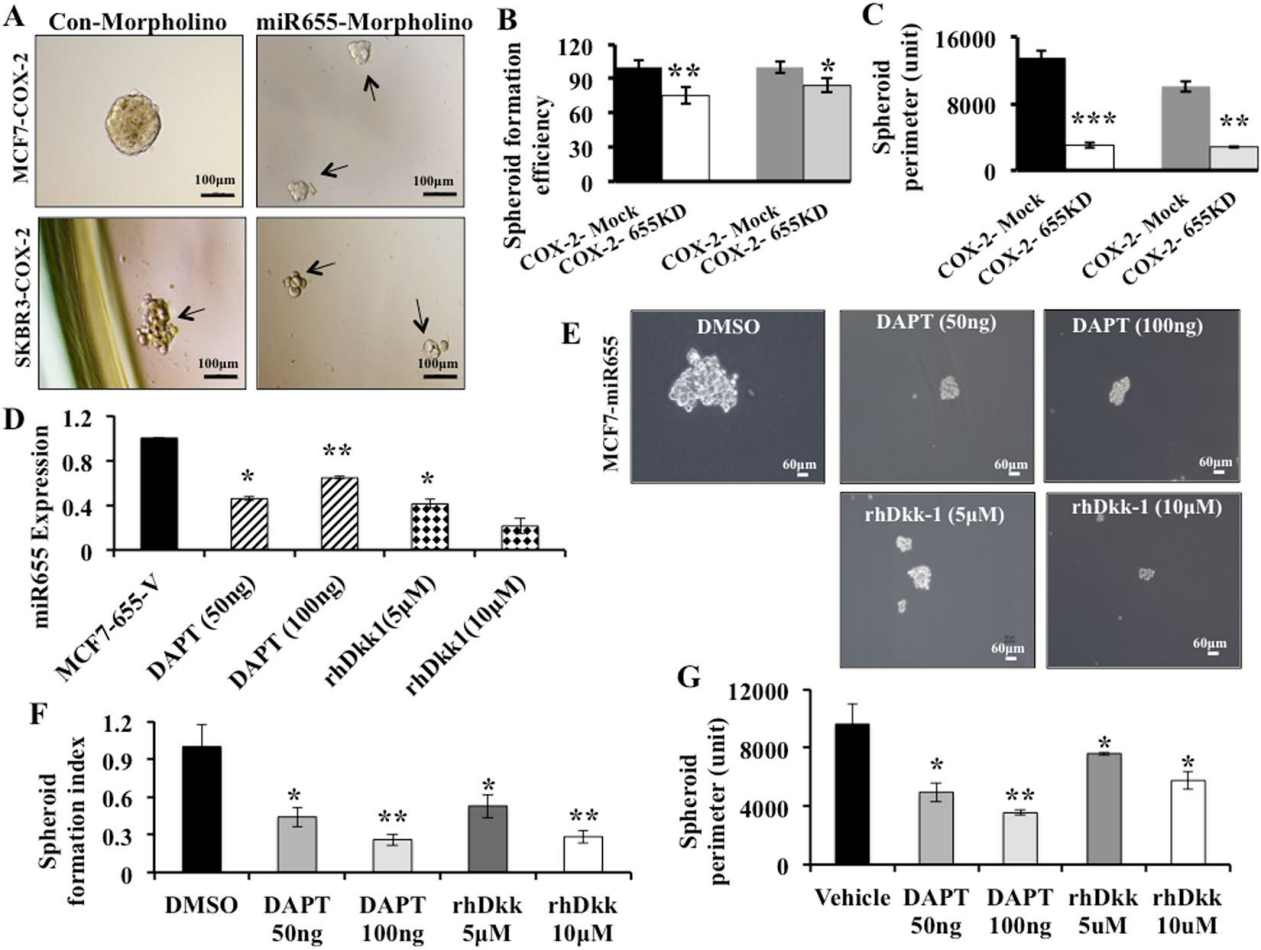
Effects of miR655 knock-down and NOCTH/WNT inhibitors on SLC phenotypes of miR655 overexpressing cells: Spheroid formation in MCF7-COX-2-655KD and SKBR3-COX-2-655KD cells (achieved with transient miR655 knock-down), compared to respective Mock controls. (**A**) Morphology, (**B**) spheroid forming efficiency and spheroid sizes (perimeter) (**C**) of MCF7-COX-2 and SKBR3-COX-2 cells after miR655 knock-down. (**D**) Expression of miR655 in MCF7-miR655 cells after treating with inhibitors of NOTCH (DAPT) and WNT (rhDkk). (**E**) Representative images for MCF7-miR655 cells after above treatments demonstrating relative spheroid number (**F**) and perimeter (**G**). Quantitative data are presented as a mean of triplicate independent experiments ± SEM. *Indicates p < 0.05, **p < 0.005, ***p < 0.001 compared to parental and mock cell lines. Scale bar is 60 µm.

Conversely, knock-down of miR655 in MCF7-COX-2 and SKBR3-COX-2 cells resulted in a significant reduction in the spheroid formation efficiency (Fig. 4A,B) and spheroid perimeter (Fig. 4C). To further link NOTCH and WNT with SLC regulation, we treated MCF7-miR655 cells with NOTCH and WNT inhibitors. Both reagents significantly reduced miR655 expression in MCF7-miR655 cells (Fig. 4D), abrogated spheroid formation, as indicated by reduced spheroid size (Fig. 4E) and numbers (Fig. 4F,G) in a dose dependent manner. These results suggest that miR655 directly promoted SLC phenotype in human breast cancer cell lines.

### MiR655 promotes lung colony formation in an experimental metastasis model

**Figure 5:**
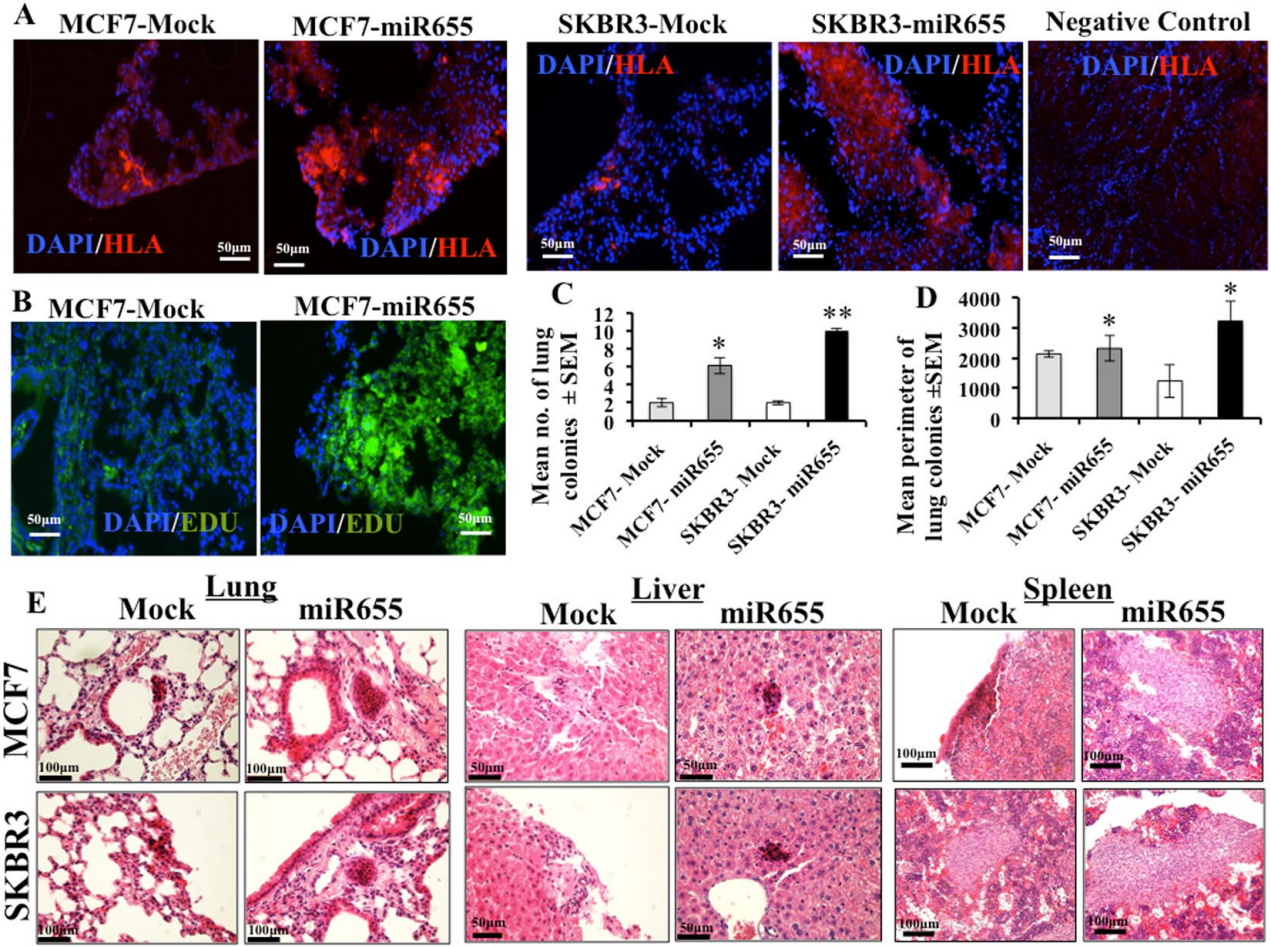
Expression of miR655 supports lung colony formation and multi-organ metastasis. Comparison of lung colony formation by MCF7-miR655 and SKBR3-miR655 cells and respective control cells in GUSB null mice, n = 5 mice per cell line. (**A**) Representative images of lung sections stained for HLA (red) and DAPI (blue) demonstrating lung colony formation 4 weeks post-injection. (**B**) Proliferative lung colonies detected with EdU staining. For (**A**) and (**B**) scale bar is 50 µm. Quantitative data of HLA staining are presented as mean of (**C**) lung colonies (>8 cells) and (**D**) perimeter. Quantitative data are presented as means of triplicate measurements ± SEM. For each lung, 10 sections were screened for colonies. *Indicates p < 0.05, **p < 0.01. (**E**) Metastatic foci in liver, spleen, and lung are detected with H&E staining. Scale bar is 100 µm.

In order to investigate possible tumorigenic functions of miR655 *in vivo*, MCF7-miR655, SKBR3-miR655 and respective Mock (control) cell lines were injected (inoculum dose of 5 × 10^5^ cells) into the tail-vein of 7-week old NOD/SCID/GUSB-null female mice and allowed to colonize for 4 weeks. Lung sections were stained with anti-Human Leukocyte Antigen (HLA) antibody to identify human tumor cells. The number of HLA-positive single cells, aggregates (2–7 cells) and colonies (8 or more cells) was counted for each of the three serial sections of the lungs in each animal using the ImageJ program. In the serial sections of the same lung we also stained for EdU, to identify proliferating tumor cells. Inocula of MCF7-miR655 cells resulted in a significantly increased size (Fig. 5A,C) and numbers (Fig. 5D) of proliferative lung colonies determined with EdU staining (Fig. 5D), compared to Mock cell lines. We screened other organs of mice for micro and macro metastasis, showing miR655 cells metastasized to the liver and the spleen (Fig. 5E). These results with an *in vivo* model of experimental metastasis revealed that over-expression of miR655 in human breast cancer cells promoted their lung colony forming ability, an oncogenic phenotype.

### Correlation of COX-2, EP4 and miR655 expression

**Figure 6:**
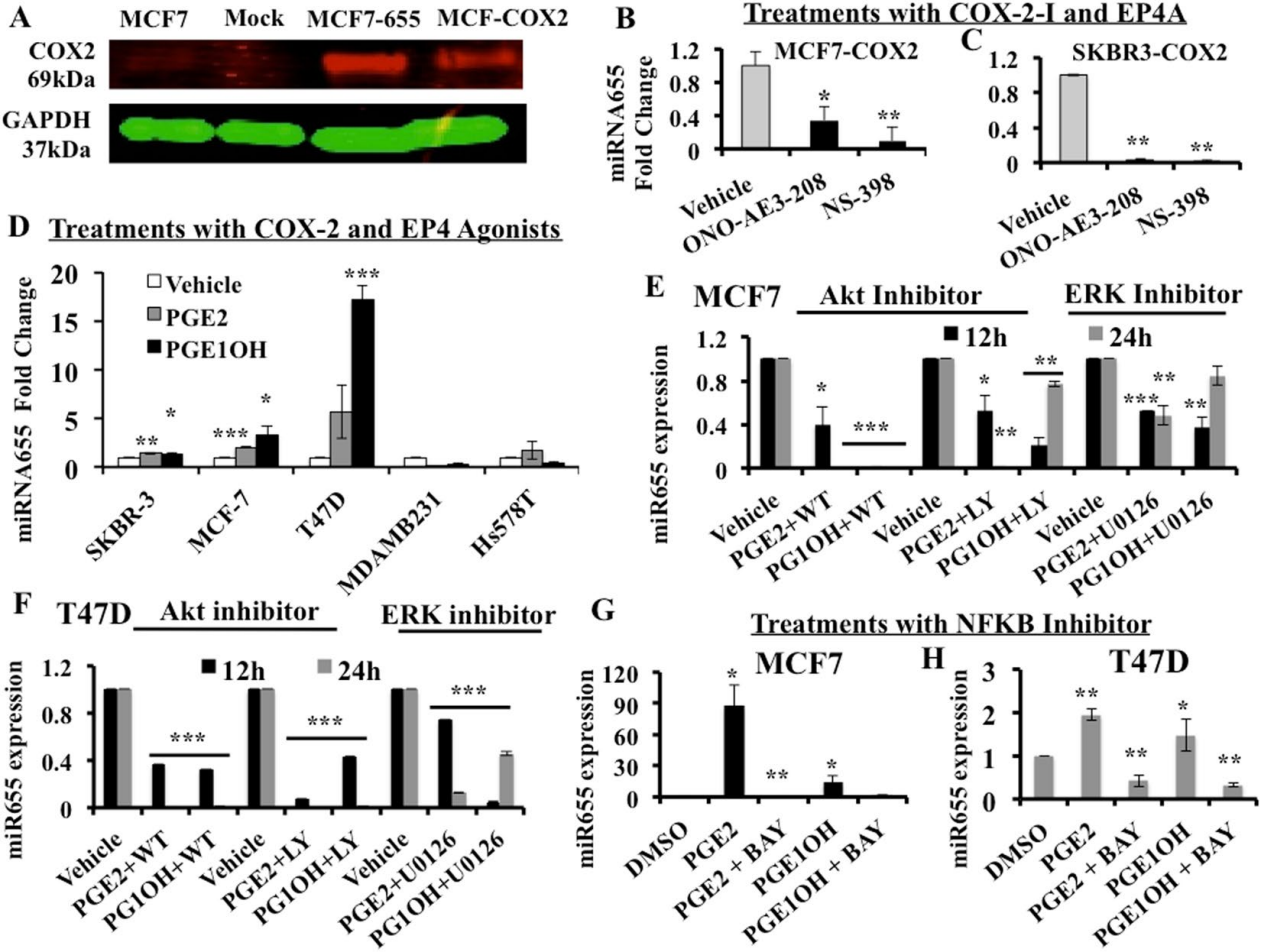
COX-2 and EP4 mediated regulation of miR655. (**A**) COX-2 protein expression in MCF7, MCF7-Mock, MCF7-COX-2 and MCF7-miR655 cells. MiR655 expression in (**B**) MCF7-COX-2 and (**C**) SKBR3-COX-2 cells treated with COX-2 inhibitor (NS-398, 10 µM) and EP4 antagonist (ONO-AE3-208, 10 µM). (**D**) Comparison of fold changes in miR655 expression relative to controls (vehicle treatment) in a panel of breast cancer cell lines treated with an EP ligand (PGE2; 10 µM), EP4 receptor agonist (PGE1OH; 10 µM), or vehicle (DMSO). Comparison of miR655 expression after stimulation with PGE2 and PGE1OH followed by treatment with two PI3K inhibitors, Wortmannin (WT), LY-240-002 (LY) both at (10 µM) and ERK inhibitor U0126 (10 µM) in (**E**) MCF7 and in (**F**) T47D cells. To test intermediary role of NF-κB in EP4 mediated regulation of miR655 in (**G**) MCF7 and (**H**) T47D cells, the cells were treated with NF-κB inhibitor BAY-11-7082 (10 µM) or vehicle (DMSO) along with PGE2 and PGE1OH. Quantitative data are presented as means of triplicate experiments ± SEM. *Indicates p < 0.05, **p < 0.01, ***p < 0.005.

In miR655 overexpressing cells we measured the levels of COX-2 protein. As shown in Fig. 6A, expression of COX-2 protein in MCF7-miR655 cells was found to be as high as in ectopic COX-2 over expressing MCF7-COX-2 cells which also expressed high EP4^23^. This finding prompted us to investigate the regulation of miR655 by COX-2 or EP4, using inhibitors and activators of COX-2 and EP4.

### Treatment with COX-2 inhibitor or EP4 receptor antagonist (EP4A) downregulates miR655 expression in COX-2 high cells

Previous studies had demonstrated that inhibiting COX-2 or EP4 receptor activity reduced proliferative and spheroid forming ability of COX-2 expressing murine C3L5 mammary cells *in vitro* ^22^, orthotopic tumor growth and spontaneous metastases *in vivo* ^18,21,22^ and the incidence of SLC marker positive cells within the residual tumors in treated mice^22^. Similarly pretreating human MCF7-COX2 with EP4A reduced their lung colony forming ability^23^. Furthermore, treatments with COX-2 inhibitors and EP4A reduced miR526b expression in human breast cancer cells^37^. To examine the potential role of the EP4 receptor in COX-2 induced miR655 expression *in vitro*, MCF7-COX-2 and SKBR3-COX-2 cells were treated with an EP4A (ONO-AE3–208), COX-2 inhibitors (NS398), or vehicles for 24 h before miRNA extraction. Both treatments caused a significant decrease in miR655 expression compared to vehicle treated cells (Fig. 6B,C). These results suggest that miR655 expression in COX-2 over-expressing cells is dependent on both COX-2 and EP4 activity.

### EP receptor activation stimulates miR655 expression

PGE2 is the major prostanoid product of COX-2 enzyme activity, and is the endogenous ligand for all EP receptors including the cAMP-stimulatory EP2 and EP4 receptors^10,11^. PGE1OH binds selectively to EP4 (signaling via cAMP and PI3K/AkT) but not EP2^12^. We treated a panel of breast (both epithelial and cancer) cell lines with PGE2 and PGE1OH for 24 h and quantified miR655 expression. While low COX-2 expressing MCF7 and T47D human breast cancer cells showed significant increases in miR655 expression with PGE2 and PGE1OH treatments (Fig. 6D), above treatments could not increase miR655 expression in MDA-MB-231 cells, which already had high expression of COX-2 and miR655.

### Treatments with AKT inhibitors (LY and WM) significantly blocks PGE2 and PGE1OH induced miRNA expression

PGE2 can bind to all EP receptors and PGE1OH is a selective EP4 agonist. To test the distinctive role of EP4 in regulating miRNA stimulation, we examined whether blocking the PI3K/AkT and ERK pathways, known to be stimulated by EP4^12–14^, but not EP2^12^, could mitigate the stimulatory effects of PGE2 and PGE1OH on miR655 expression. We first treated MCF7 and T47D cells with PGE2 or PGE1OH, followed by one of two PI3K inhibitors LY and WT and ERK inhibitor U0126 for 12 h and 24 h. Following miRNA extraction and qPCR analysis, we observed that treatment with both PI3K inhibitors significantly blocked miR655 up-regulation in a time dependant manner in both cell lines. ERK inhibitor could also abrogate miR655 expression (Fig. 6E,F). These results reveal the role of PI3K/AkT and ERK signalling, presumably mediated by EP4 activation, in stimulating miR655 expression in MCF7 human breast cancer cells. However, we could not exclude the role of cAMP/PKA pathway shared by EP4 and EP2.

### Role of NF-κB in miR655 regulation

Surprisingly, we observed that miR655 expression leads to COX-2 up-regulation. MiR655 over expression increased COX-2 protein expression (Fig. 6A). Conversely, following miR655 knockdown in MC7-COX-2 and SKBR3-COX-2 cells, COX-2 protein was significantly downregulated (Supplementary Figure 2). NF-κB is known to play an important role in up-regulating COX-2 in a variety of human cancers^51^ and we have shown that miR526b is regulated by COX-2/EP4 via NF-κB pathway^37^. So, we tested whether NF-κB played an intermediary role in COX2/EP4 mediated miR655 upregulation.

**Figure 7:**
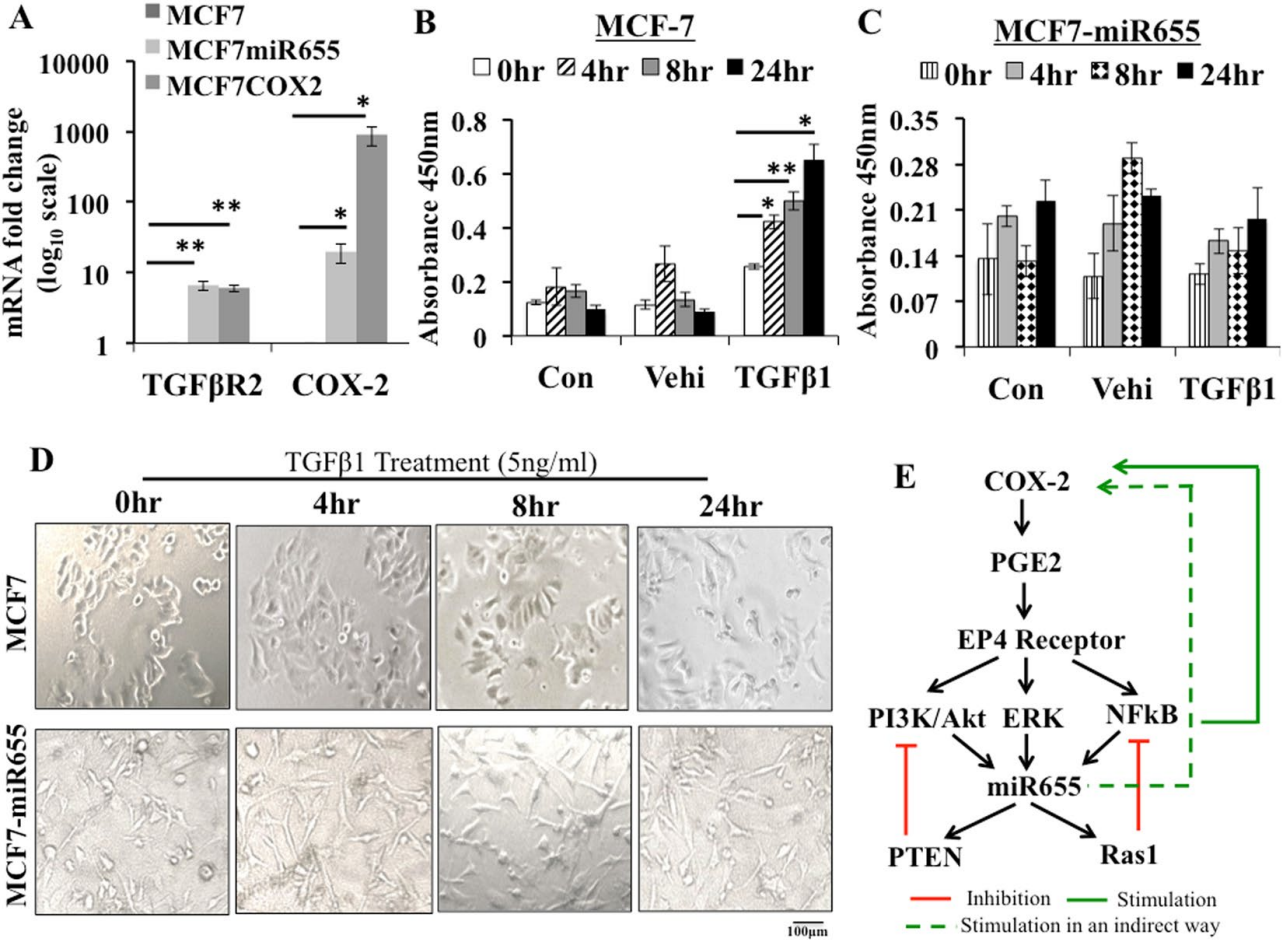
MiR655 expression results in TGFβ resistance. (**A**) Both MCF7-miR655 and MCF7-COX2 cells show significantly high expression of TGFβR2 and high expression of COX-2 mRNA compared to MCF7 cells. We treated all three cell lines with TGFβ1 (5 ng/ml) and collected cell lysates at various time intervals 0, 8, 12 and 24 hrs and measured pSmad3 with InstantOne™ ELISA. Phosphorylated SMAD3 (Ser423/425) was measured in cell lysates by absorption values at 450 nm. (**B**) pSmad3 was significantly upregulated in MCF7; (**C**) but no change was noted in MCF7-miR655 cells. (**D**) MCF7 cell morphology changed with TGF-β1 treatment from epitheloid to fibroblast-like, however MCF7-miR655 cell morphology (fibroblast-like) did not change. (**E**) Schema represents possible regulation of miR655 by COX-2 and vice-versa.

We identified tumor suppressor gene *TP53NP1* (tumor protein p53 inducible nuclear protein 1) gene, which is a negative regulator of NF-κB, is significantly down regulated MCF7-COX-2 (Supplementary Table 1) in miRNA target gene search. We also conducted post-study genome data mining to search miR655 target genes with MIRBASE and MIRDB softwares to identify another NF-κB negative regulator *RAB7L1* (RAS oncogene family-like 1) gene. A down-regulation of *RAB7L1* or *TP53NP1* could thus lead to up-regulation of NF-κB, and subsequent up-regulation of COX-2 and EP4. *PTEN* a negative regulator of PI3K, one of the genes downregulated in the same MCF7-COX-2 cells (Supplementary data in ref.^23^), in which we identified miR655; suggesting a possible mechanism of PTEN down-regulation leading to up-regulation of PI3K/AkT signaling via the EP4 receptor. To test the role of NF-κB in miR655 regulation, we treated MCF7 and T47D cells with BAY-11-7082 (10 μM), a NF-κB inhibitor along with PGE2 and PGE1OH for 24 h. BAY-11-7082 significantly blocked PGE2 and PGE1OH stimulated miR655 expression in both MCF7 and T47D cells (Fig. 6G and H respectively), indicating NF-κB is an intermediary in miR655 upregulation by EP4. We interpreted these results representing a positive feedback loop for COX-2/EP4/NF-κB/miR655/COX-2-mediated SLC perpetuation (schema presented in Fig. 7E).

### MiR655 over-expression makes cells refractory to TGF-β mediated stimulation of Smad3 phosphorylation

It has been shown that human breast cancer can evolve from a TGF- β sensitive to a TGF-β resistant stage, and one of the mechanisms is activation of COX-2/EP4 pathway, making the cells unresponsive to TGF-β mediated phosphorylation of Smad3^52^. We investigated whether discrepancies between our results of miR655 as an EMT promoter in miR655 over-expressing breast cancer cells and other reports of miR655 being an EMT suppressor in cancer cell lines exhibiting TGF-β-induced EMT^43^ could be explained by subversion to COX2/EP4 pathway. We compared the effects of TGF-β treatment on MCF7 cells (low COX-2, low miR655) with those on miR-655 overexpressing MCF7-miR655 cells, and measured level of Smad-3 phosphorylation (Fig. 7). Our contention was supported by several findings: (1) MCF-7-miR665 cells exhibited 8–10 fold upregulation of TGFβ-R2 relative to MCF-7 cells (Fig. 7A), rather than miR655 mediated downregulation of TGFβ-R2 as noted by Harazano *et al*.^43^. (2) MCF7 cells exhibited a time-dependent increase in Smad-3 phosphorylation, as expected (Fig. 7B) when treated with TGF-β1 (5 ng/ml) and also exhibited a mesenchymal phenotype (fibroblast-like morphology) (Fig. 7D). In contrast MCF7-miR665 cells exhibited no change in Smad-3 phosphorylation or in morphology when treated with TGF-β1 (Fig. 7C,D).

### MiR655 expression in human breast tissues and plasmas and correlation with overall patient survival

**Figure 8:**
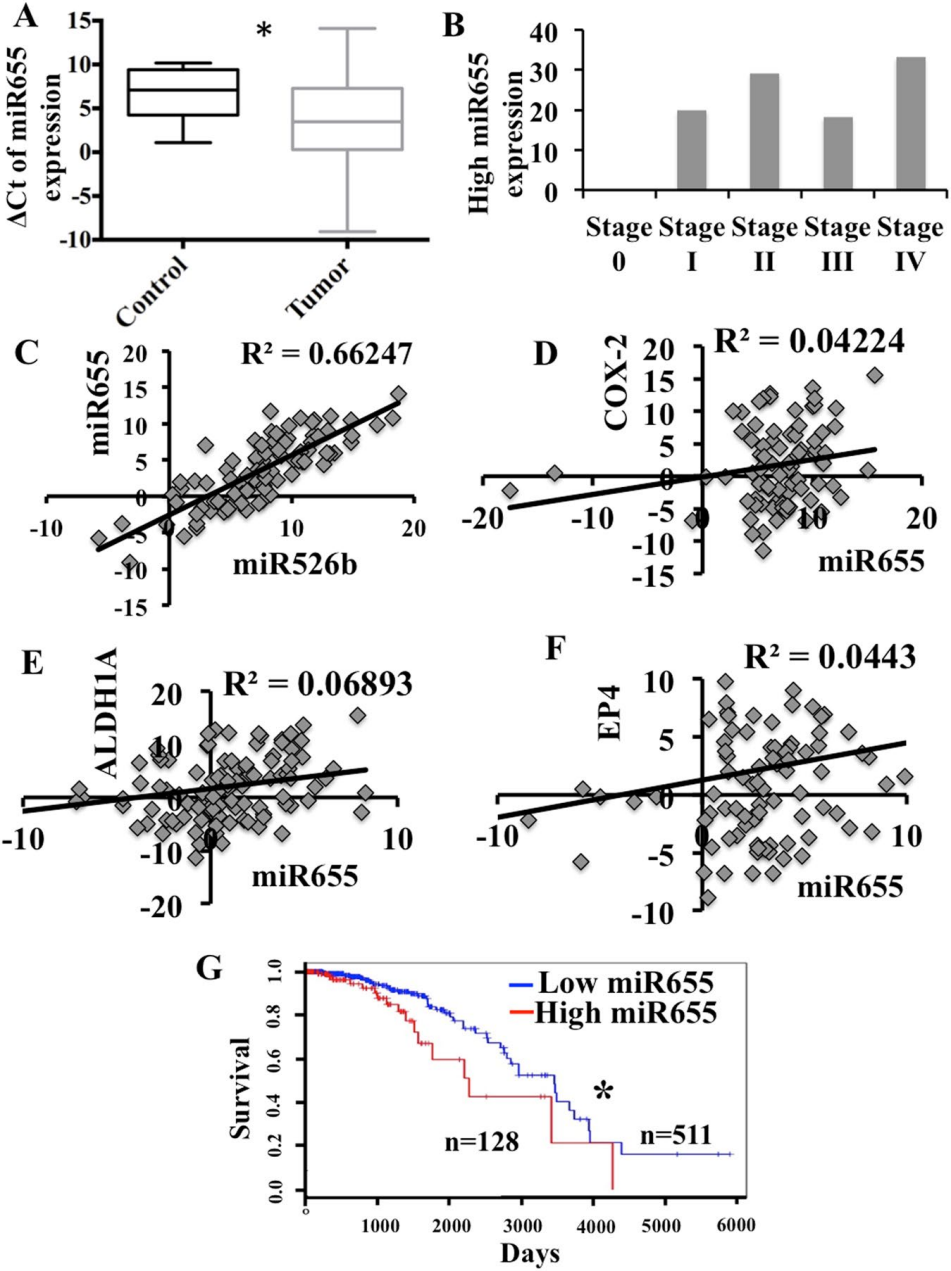
MiR655 is over-expressed in breast tumors and negatively correlates with patient survival. (**A**) Box-and-whisker plot showing over-expression of miR655 in primary human breast tumors (n = 105), in comparison to non-tumor control tissue (n = 20). A more negative ΔCt value indicates a higher miRNA expression level. (**B**) Expression of miR655 in different stages of tumors. Very significant correlation exists between (**C**) miR526b and miR655 expression. There is a positive correlation between certain genes and miRNA expression, such as between (**D**) miR655 and *COX2*, (**E**) miR655 and *EP4* and (**F**) stem cell marker *ALDH* and mir655 expression. (**G**) Kaplan-Meier curve demonstrating the inverse relationship between high miR655 expression and overall survival rates in primary human breast carcinomas (n = 511). *Indicates p < 0.01.

A comparison of miR655 expresssion in 105 primary breast cancer and 20 adjacent non-cancerous tissues revealed significantly higher expression in cancerous tissues (Fig. 8A). Expression of miR655 was observed in all stages of tumor except stage 0 (Fig. 8B). A very strong positive correlation between miR526b and miR655 expression was also observed in tumor tissues (Fig. 8C). A weak but positive correlation between miRNA655 and *COX-2, EP4* and SLC marker *ALDH1A* expression in tumor tissues were noted; their expression was also higher than non-cancerous tissues (Fig. 8D–F). The lack of a more robust correlation can be explained by the likelihood that levels of COX-2 or EP4 or ALDH mRNA may not reflect COX-2 or EP4 and ALDH activity earlier shown to upregulate this miRNA. Cancer genome atlas data mining revealed that high miR655 expression (80th percentile) in breast cancers is associated with decreased overall survival (Fig. 8G).

## Discussion

COX-2 is a major driver of human breast cancer progression and metastasis^2–6^ via activation of the PGE receptor EP4^18,19,21,22^, leading to SLC-stimulation^22,23^ and induction of an oncogenic and SLC-promoting miRNA miR526b^37^. In the present study, we show that both COX-2 and EP4 activities directly upregulated another oncogenic and SLC-stimulating miRNA miR655 in human breast cancer. In aggressive COX-2 expressing breast cancer cell lines, treatments with EP4A and COX-2 inhibitor could reduce miR655 expression. Our results further suggest a positive feedback-regulatory loop for SLC promotion/sustenance via COX-2/EP4/NF-κB/miR655/COX-2 axes. High expression of miR655 was detectable in higher-grade primary human breast cancer tissues and negatively correlated with overall patient survival. These findings reveal an association of miR655 expression with disease progression, and its potentials as a biomarker in breast cancer patients.

In surprising contrast to our findings, miR655 was reported as an EMT-suppressive miRNA targeting ZEB1 and TGFBR2 in pancreatic cancer cell lines^43^. In esophageal squamous cell carcinoma cell lines, miR655 over-expression was reported to suppress cellular invasion by targeting pituitary tumor-transforming gene-1^44^. Furthermore, miR655 was reported to suppress EMT by targeting Prrx1 in triple-negative breast cancer (TNBC)^45^. These authors reported that miR655 expression was significantly lower in TNBC compared to control tissues, in sharp contradiction to our findings noted above. While we cannot provide a simple reason for these contradictory findings in human breast cancer, we suggest that functions of any miRNA are context-dependent^53^. This is because of (a) multiplicity of target genes, some of which may have opposing functions; (b) mRNA profiling to determine microRNA targets may still miss relevant target genes; (c) multiplicity of upstream regulators of each miRNA. Upstream signaling initiates transcription of miRNA genes and may also create feedback loops by targeting their own transcription factor(s); (d) targets genes may resist a particular miRNA action owing to their subversion by other genes/pathways. We believe that miR655 is no exception to the complexity of determinants of miRNA actions listed above. For example, miR655 was shown to suppress TGF-β-induced EMT in pancreatic cancer cells^43^ which depended on Smad 2/3 activation, whereas miR655 mediated promotion of EMT observed in the present study is due to COX-2/EP4 activation. It was shown that COX-2 inactivates Smad signaling and enhances EMT stimulated by TGF-β through a PGE2/EP receptor dependent mechanism^52^. This pathway was shown to uncouple TGF-β from Smad 2/3 signaling in COX-2 expressing breast cancer cells explaining the switch of TGF-β from a tumor-suppressor to a tumor-promoter during mammary carcinogenesis^52^. We show here that parental or Mock-transfected MCF7 cells responded to exogenous TGFβ1 by assumption of mesenchymal morphology and increased Smad3 phosphorylation, whereas MCF7-miR655 cells exhibited EMT phenotype and were refractory to TGFβ1-mediated Smad3 phosphorylation. We have further established that the PGE2/EP4 mediated signaling via PI3K/AkT and ERK pathways, which promotes cell survival and migration, is responsible for miR655 upregulation. Thus the discordance of miR655 mediated EMT responses shown in the present study from earlier reports^43,45^ can be explained in the context of COX-2/EP4 mediated signaling. Finally, our results suggest COX-2/EP4/NF-κB/miR655/COX-2 axis mediates SLC perpetuation. The proposed schema for signaling mechanisms is shown in Fig. 7E.

MiRNA profiling has been utilized in a large number of human breast cancer cell lines to identify tumor subtypes and driver mutations^54^. We show that COX-2 overexpression in breast cancer cell lines induced the expression of two oncogenic and SLC-promoting miRNAs, miR526b^37^ and miR655 (present study). In our combined miRNA and gene micro arrays we identified 13-tumor suppressor like genes targeted by these two miRNAs, down regulated in MCF7-COX-2 cells^37^. We show that expression of both miRNAs were positively correlated with each other in human breast cancer tissues, higher in high-grade breast cancer, and associated with reduced patient survival. These miRNAs add to the list of other oncogenic miRNAs in breast cancer, such as miR-106b-25 cluster^55^, miR-10b^56^, miR-21^57^, miR-9^58^ shown to be associated with one or more events in tumor progression and metastasis. Amongst these, miR-9 has been indirectly linked with SLC phenotype^59^. On the other hand, expression levels of *let-*7 miRNAs were shown to correlate inversely with SLC stimulation in human breast cancer. Forced over-expression of *let-*7 miRNAs in human breast cancer cell lines resulted in a reduction in SLC marker bearing cells as well as a spheroid forming ability^40^. Down-regulation of the *let-*7 family of miRNAs could distinguish SLC populations from non-SLC populations within primary breast tumors^40^. However, none of the above-listed oncogenic or anti-oncogenic miRNAs have yet been directly linked with SLC functions. In the present study, we provide multiple evidences for miR655 being directly associated with SLC stimulation. We showed that ectopic over-expression of miR655 in human breast cancer cell lines markedly increased their ability to form spheroids *in vitro*, induced NOTCH gene expression, and increased the expression of SLC markers ALDH, OCT3/4 and lung colony forming ability *in vivo*. Furthermore, both NOTCH and WNT inhibitors significantly reduced spheroid formation by miR655 overexpressing cells. We suggest that miR655 mediated upregulation of COX-2 is one mechanism explaining SLC stimulation by this miRNA.

In conclusion, our results taken together established that miR655 is an oncogenic miRNA in human breast cancer, promoting EMT, cellular migration, invasion, proliferation, SLC stimulation and metastasis *in vivo*. The oncogenic functions resulted from EP4 activation and PI3K/AkT and ERK signaling, promoting cell survival and migration and upregulation of NOTCH associated with SLC phenotype. MiRNA-mediated downregulation of NF-κB could explain COX-2 upregulation to sustain SLC phenotype.

## Materials and Methods

### Ethics Statements

The human breast cancer and the adjacent non-cancerous tissues used in this study were obtained from the Ontario Institute for Cancer Research (OICR) repository (Ontario Tumor Bank, Toronto, CA) based on approval by the University of Western Ontario Human Research Ethics Board. Sample processing, RNA and miRNA extraction and cDNA synthesis were performed in accordance with the relevant guidelines and regulations of University of Western Ontario. We conducted experiments at the Brandon University, following the regulations of Brandon University Research Ethics and Biohazard Committee. Use of mice was approved by the Animal Use Subcommittee of this University of Western Ontario, according to the guidelines of the Canadian Council on Animal Care.

### Cell lines

All human breast cancer cell lines (MCF7, SKBR3, MDA-MB-231 and T47D) were purchased from American Type Culture Collection (ATCC, Rockville, MD). Stable COX-2 over expressing MCF7-COX-2 and SKBR3-COX-2 cell lines was generated in our laboratory as described previously^21^. MCF7 and MCF7-COX-2 cells were grown in Eagle’s Minimum Essential Medium (EMEM), supplemented with 0.4 µl/mL human insulin (ATCC). SKBR3 and SKBR3-COX-2 were grown in McCoy’s 5 A Modified Medium with L-glutamine (GIBCO, ON). MDA-MB-231 (high COX-2, ER and HER-2 negative) and T47D (low COX-2, ER positive and HER-2 negative) were grown in RPMI-1640 minimal essential medium (GIBCO, ON). All media were supplemented with 10% fetal bovine serum (FBS) and 100 U/mL penicillin and 100 µg/mL streptomycin (Sigma, ON) and maintained in a humidified incubator with 5% CO2 at 37 °C. Additionally MCF7-COX-2 and SKBR3-COX-2 and their respective mock cell lines maintained with Geneticin® (GIBCO, ON) at 500 μg/mL.

### MiRNA micro array

We conducted miRNA micro arrays (quadruplicate measurements) comparing miRNA expression changes in MCF7-COX-2 and MCF7-Mock control cells, using Affymatrix Genechip miRNA Array as per the manufacturer’s protocol. ANOVA with a nominal alpha value set to 0.05 was then used to identify significant changes, followed by Benjamini-Hochberg multiple testing correction in order to reduce the false positive rate. These results were then separated into significant increases or decreases, and used in a cross-platform analysis.

### Drugs and reagents

NS398 (COX-2 inhibitor) was purchased from Cayman Chemical (Michigan, MI). ONO-AE3-208 (selective EP4 antagonist) was a gift of ONO Pharmaceuticals, Osaka, Japan. PGE2 (EP1-4 ligand) and PGE1OH (EP4 agonist) were purchased from Cayman Chemical. Wortmannin (WM), an irreversible PI3K inhibitor, and LY-204002 (LY), a reversible PI3K inhibitor all purchased from Sigma-Aldrich, ERK1/2 inhibitor U0126 from Cell Signalling, MA. NF-κB inhibitor, Bay 11-7082 (Sigma-Aldrich, ON, Cat # B5556) is a kind gift of Dr. Xiufen Zheng (Department of Pathology and Laboratory Medicine, University of Western Ontario). For all treatments *in vitro* or *in vivo*, respective vehicles served as the control.

### Real-time PCR

Total RNAs were extracted using miRNeasy Mini Kit (Qiagen, ON) and reverse transcribed using the TaqMan microRNA and mRNA cDNA Reverse Transcription Kit (Applied Biosystems, ON). The TaqMan MiRNA or Gene Expression Assays was used for quantitative PCR. RNU44 or RNU48 was considered as internal control miRNAs. Expression for *COX-2, E-Cadherin, ZEB1, Vimentin, SNAIL*, *TWIST, NOTCH1-3, AXIN1, 2, MYC, CCDN1, COX-2, ALDH1A, EP4* and *TGFβR2* were normalized to the values of *GAPDH* and *RLP5* control genes expression.

### Western blot

Cells were treated with M-PER® Mammalian Protein Extraction Reagent supplemented with HALT Protease Inhibitor Cocktail and Phosphatase Inhibitor Cocktail (Thermo Scientific, Rockford, IL) to extract protein. Twenty micrograms of total protein were electrophoresed per well on a SDS-polyacrylamide gel and transferred onto Immobilon-FL PVDF membranes (Millipore, Billerica, MA). Membranes were then incubated with the following primary antibodies: E-Cadherin (Cell Signaling, cat # 3195 S); Vimentin (Millipore, cat # MAB3400); TWIST1 (Santa Cruz, cat # sc15393), N-Cadherin from Cell Signaling (cat # 4061) and β-Actin (Santa Cruz, cat # sc47778) and probed with a mixture of IRDye polyclonal secondary antibodies (LI-COR Biosciences, Lincoln, NE). Images were read with an Odyssey infrared imaging system (LI-COR Biosciences).

### Immunofluorescence

Cells were grown on glass coverslips to 70–80% confluency. The cells were fixed in 4% paraformaldehyde, then permeablized in 0.5% Triton-X-100, blocked with 8% BSA + 0.01% Tween 20 in PBS and incubated in primary antibody at the following dilutions in 4% BSA overnight at 4 *°*C: E-Cadherin and Vimentin (Cell Signalling, 1:500), TWIST1 in 1:200 ration (Santa Cruz, Dallas, TX), ALDH1A and OCT3/4 (both 1:300 dilution) from BD biosciences. The stained cells were incubated with secondary antibody (1:500, Biotium, ON). Vectashield anti-fade mounting medium with DAPI (Vector) was used to mount the slides. Immunofluorescent images were taken using the Zeiss LSM 510 Meta Multiphoton Confocal Microscope.

### Stable miRNA knock-in

MCF7 and SKBR3 cells were transfected with 2 µg of either pCMV-MIR Mock vector (control) or pCMV-MIR miR655 expression plasmids (OriGene, MD) using the Amaxa Cell Line Nucleofector Kit V (Lonza, MO), according to the manufacturer’s protocol. Transfected cells were selected with the Geneticin® (GIBCO) at 500 μg/ml treatment for 3 weeks post transfection. MCF7 and SKBR3 cell lines stably transfected with the pCMV-MIR-Mock (empty) vector were referred to as MCF7-Mock and SKBR3-Mock respectively, and transfected with the pCMV-MIR miR-655 expression plasmid were referred to as MCF7-miR655 and SKBR3-miR655. All four-cell lines were maintained with low dose of Geneticin (500 ng/ml) in regular growth media.

### Transient miRNA knock-down

We transfected MCF7-COX-2 and SKBR3-COX-2 cell lines with morpholino oligonucleotides MO^655^ (20 μM) to knock-down miRNAs (Gene Tools LLC, Philomath, OR)^60^ following manufacturer’s protocol (Lonza, MO). Functional assays as described later (migration, invasion, proliferation and spheroid formation) were performed within 48 h post transfection.

### Migration, Invasion and Proliferation assays

For migration assays, 6 × 10^4^ cells in 300 µl basal media were added to the upper chamber of transwells including a multi-porous polycarbonate membrane (8 µm pore size) insert (Corning Costar Corporation, MA), and placed in a 24-well plate (BD Falcon, CA). For invasion assays, cell inserts were coated with Matrigel (1:100 in basal media; BD Biosciences, CA). The lower chamber contained 700 µl of either serum-free media or 2% FBS-supplemented media. Cells completing migration at 24 h or invasion at 48 h at 37 °C were quantified as reported earlier^18–23^. BrdU ELISA (Roche Applied Science, IN) was used to measure proliferation of MCF7 and MCF7-derived cell lines at 6 h, and SKBR3 and SKBR3-derived cell lines at 8 h^23,47^.

### Spheroid formation

Cells were plated on ultra-low attachment plates (Corning, MA) at dilution of 1 cell/100 μl in 96 well plates or 2 × 10^4^ cells/2 ml in 6 well plates with spheroid culture media as previously described^22,23,37^. Images of spheroid were captured with a light microscope and the number and perimeter of spheroids calculated using ImageJ. Spheroids were harvested and RNA extracted to conduct real-time qPCR for target gene expression.

### Lung colony assay

Seven-weeks-old female NOD/SCID/GUSB (Glucorunidase-beta)-null mice (Robarts Research Inst., London, ON) were treated in accordance with the guidelines set by the Canadian Council on Animal Care. Animals were given a tail vein injection of an inoculum dose of 5 × 10^5^ cells, and sacrificed after 4 weeks to assess micro-metastases to the lung. We excised lungs, livers, spleens, kidneys and brains and cut into two pieces, one frozen for immuno-histochemical analysis of HLA and another fixed for H&E staining. Lungs were harvested after inflation with PBS. At least 3 semi-serial 10 µm thick sections of these organs from each animal were stained with mouse anti-human HLA antibody (1:100 dilution, Sigma-Aldrich, ON) to detect tumor cells^23,37^. Proliferating cells in lung colonies were identified with Alexa Fluor conjugated EdU (Invitrogen, ON) tail vain injection 12 hrs before the mouse sacrifice.

Serial sections of all organs were imaged using a fluorescence microscope and the numbers of HLA stained lung colonies formed (8 or more cells) were calculated. Negative controls were provided by an equivalent concentration of mouse Ig iso-type replacing the primary antibody to exclude nonspecific staining. Although NOD/SCID/GUSB hosts were chosen initially to identify tumor cells in the lungs by staining for the GUSB marker, we adopted HLA staining preferred over GUSB staining since our preliminary studies revealed that some human cancer cells lost GUSB staining as lung colonies grew bigger^37^.

### TGFβ treatment and pSmad3 ELISA

MCF7 and MCF7-miR655 cells were grown in regular growth medium in 96 well plates until confluent. Then cells were serum starved for 8 h and treated with either 0.1% BSA (Sigma, ON) as Vehicle or TGFβ1 (5 ng/ml) (ThermoFisher Scientific). Then at 4, 8 and 24 hrs we captured images of cells with an inverted microscope with respective treatments and then lysed cells in the 96 well plates and processed for enzyme-linked immunosorbent assay (ELISA) following InstantOne™ ELISA (ThermoFisher Scientific) protocol to measure phosphorylation of Smad3 (pS423/pS425) with absorbance at 450 nm measured with FilterMax F5 multi-plate reader (Molecular Devices, Sunnyvale, CA).

### Human tissue

To examine the clinical relevance of miR655 expression in breast cancer, we obtained frozen female human breast tumor (n = 105) and control (n = 20) tissues (adjacent non-tumor tissue from unrelated patients) from the Ontario Tumor Bank, maintained by the Institute for Cancer Research (OICR). Demographic description of the sample set is described earlier^23,37^. We used Qiagen RNA and miRNA extraction kits to extract mRNA/miRNA from tissue followed by cDNA synthesis and Taqman miRNA and gene expression assays (miR655, *COX-2, EP4* and *ALDH1A*) as described before^23,37^. Negative ∆Ct values (≤0) are indicative of higher miRNA expression in tissue samples.

### Survival analyses

Coded patient survival data was extracted from the TCGA clinical information file. Patient survival was calculated as time in months elapsed from date of diagnosis until date of last contact. Kaplan-Meier curves for overall survival associated with miR655 expression were conducted in 639 primary breast carcinomas. A cut off p value (p < 0.05) was determined using log rank test.

### Statistical analysis

Statistical calculations were performed using GraphPad Prism software version 5 (GraphPad Software, CA). All parametric data were analyzed with one-way ANOVA followed by Tukey-Kramer or Dunnett post-hoc comparisons. Lung colony numbers were analyzed both by parametric and non-parametric (Wilcoxon rank sum test) methods, giving same results. Student’s t-test was used when comparing two datasets and Pearson’s coefficient was employed to assess statistical correlations. Statistically relevant differences between means were accepted at p < 0.05.

## Electronic supplementary material

Supplementary Material
