# Supplementary material for: COX-2 induces oncogenic micro RNA miR655 in human breast cancer
Source: Sci Rep. 2018 Jan 10;8:327. doi: 10.1038/s41598-017-18612-3 (PMC5762661; doi:10.1038/s41598-017-18612-3)
Supplement: Supplementary file 1 — Supplementary Material [file 41598_2017_18612_MOESM1_ESM.pdf]

Title: COX-2 induces oncogenic micro RNA miR655 in human breast cancer

Authors: Mousumi Majumder, Leanna Dunn, Ling Liu, Asma Hasan, Krista Vincent, Muriel Brackstone, David Hess, Peeyush K Lala.

Corresponding author: Dr. Peeyush K. Lala (pklala@uwo.ca), Department of Anatomy and Cell Biology, University of Western Ontario, 1151 Richmond St., London, Ontario, N6A 5C1, Canada

Supplementary Figure Legends:

Supplementary Fig 1. The data are presented as a mean of triplicates ± SEM. * Indicates $p<0.05$. Taqman expression assay of (A) miR655 compared to RNU44 miRNA in all breast cancer cell lines. (B) MCF7 and SKBR3 cells transfected with the miR655 expression plasmid displayed approximate 10,000-fold increase in miR655 expression compared to empty vector (Mock) control cells. (C) Transient transfection of MCF7-COX-2 and SKBR3-COX-2 cells with 20 µm of miR-655-specific morpholinos displayed approximate 40-fold and 60-fold decreases, respectively, in miR655 expression compared to empty vector (Mock) control cells.

Supplementary Fig 2: Western blot analyses of COX-2 in MCF7-COX-2 and COX-2 high cells after miR655 knock down. MDAMB231 cell line served as positive control.

Supplementary Fig 3: Western blot analysis of Vimentin in MCF7 and MCF7-derived cell lines. MDAMB231 cell line served as positive control.

Supplementary Fig 4: Western blot analysis of E-Cadherin in MCF7 and MCF7-derived cell lines. MCF7 cell line served as positive control.

Supplementary Table 1: miR655 target gene list. We tabulated only down regulated genes.

Supplementary Fig 1

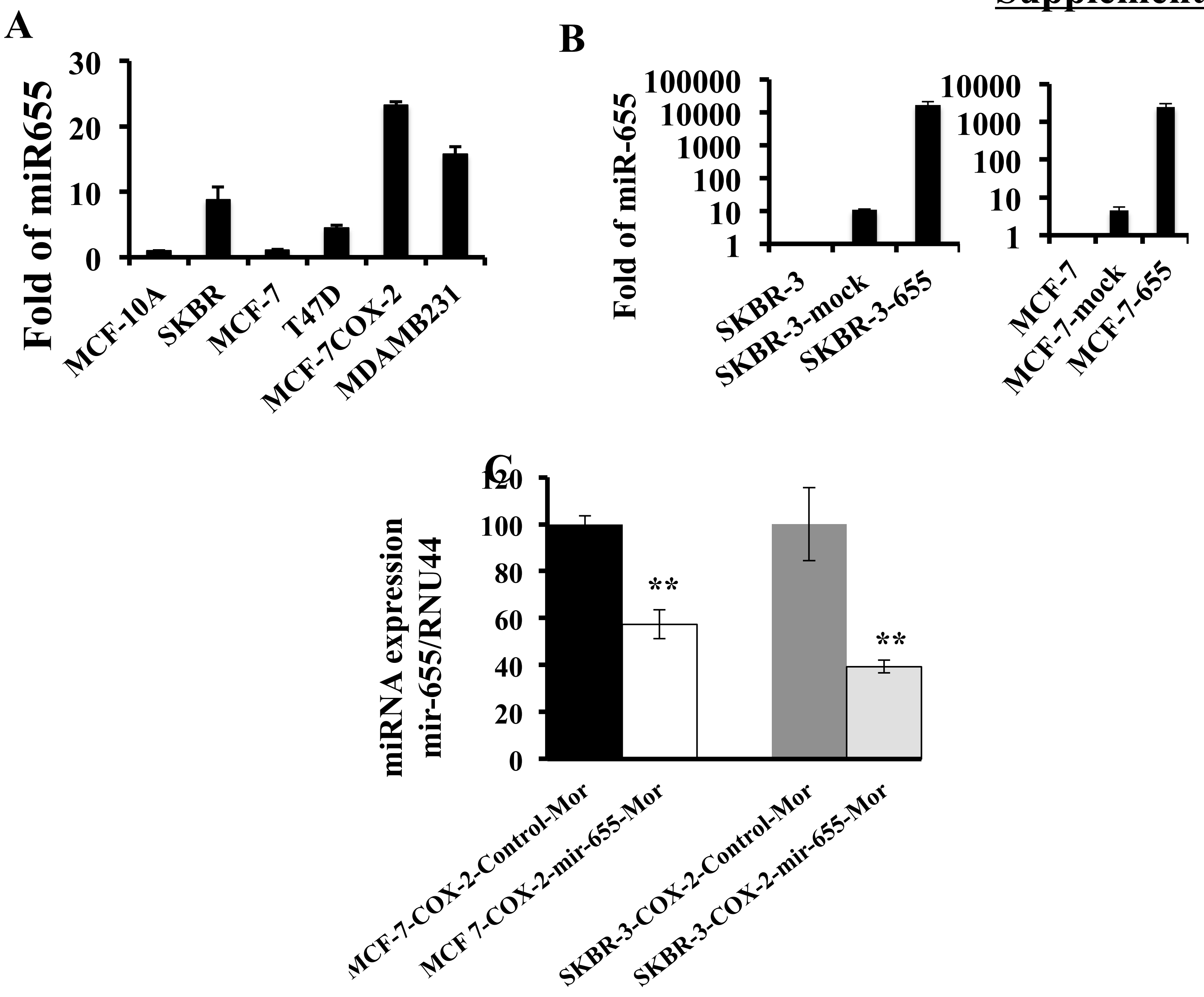

Supplementary Figure 2

COX-2 Western Blot

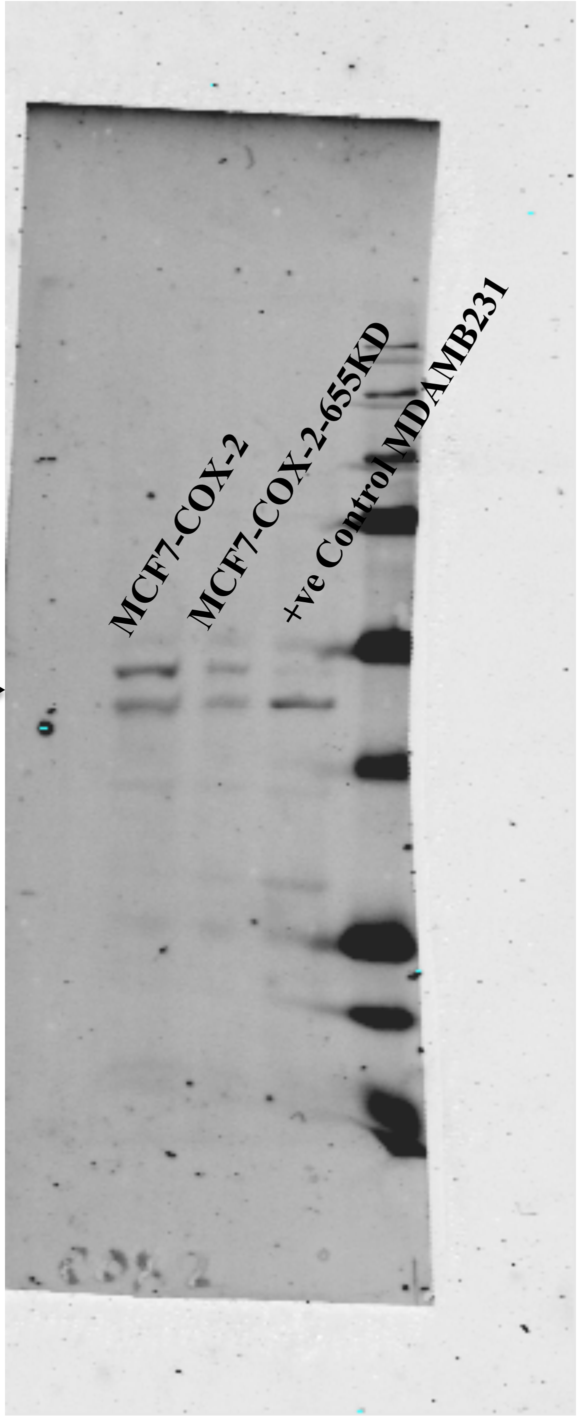

COX-2 →

Supplementary Figure 3

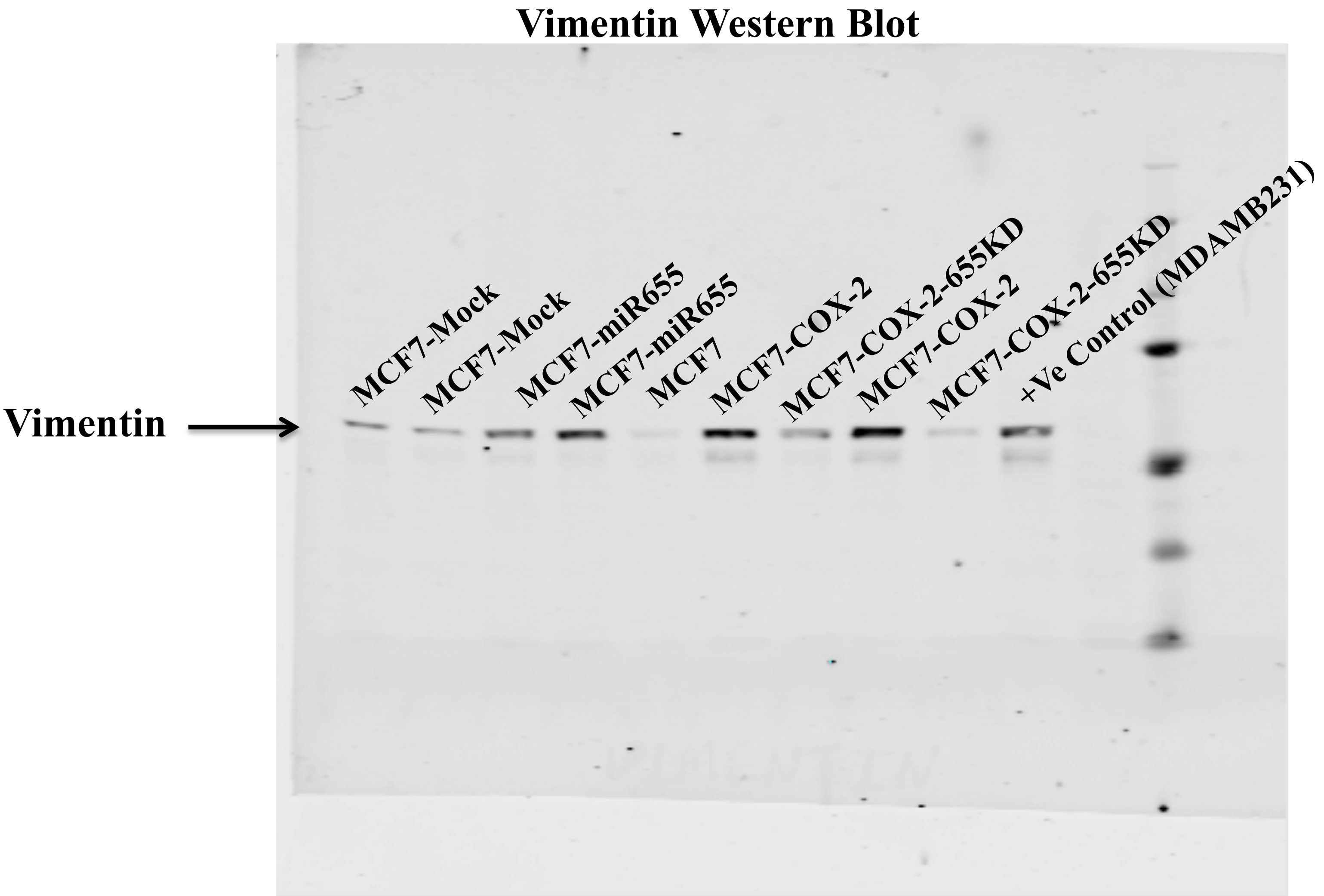

Supplementary Figure 4

E-Cadherin Western Blot

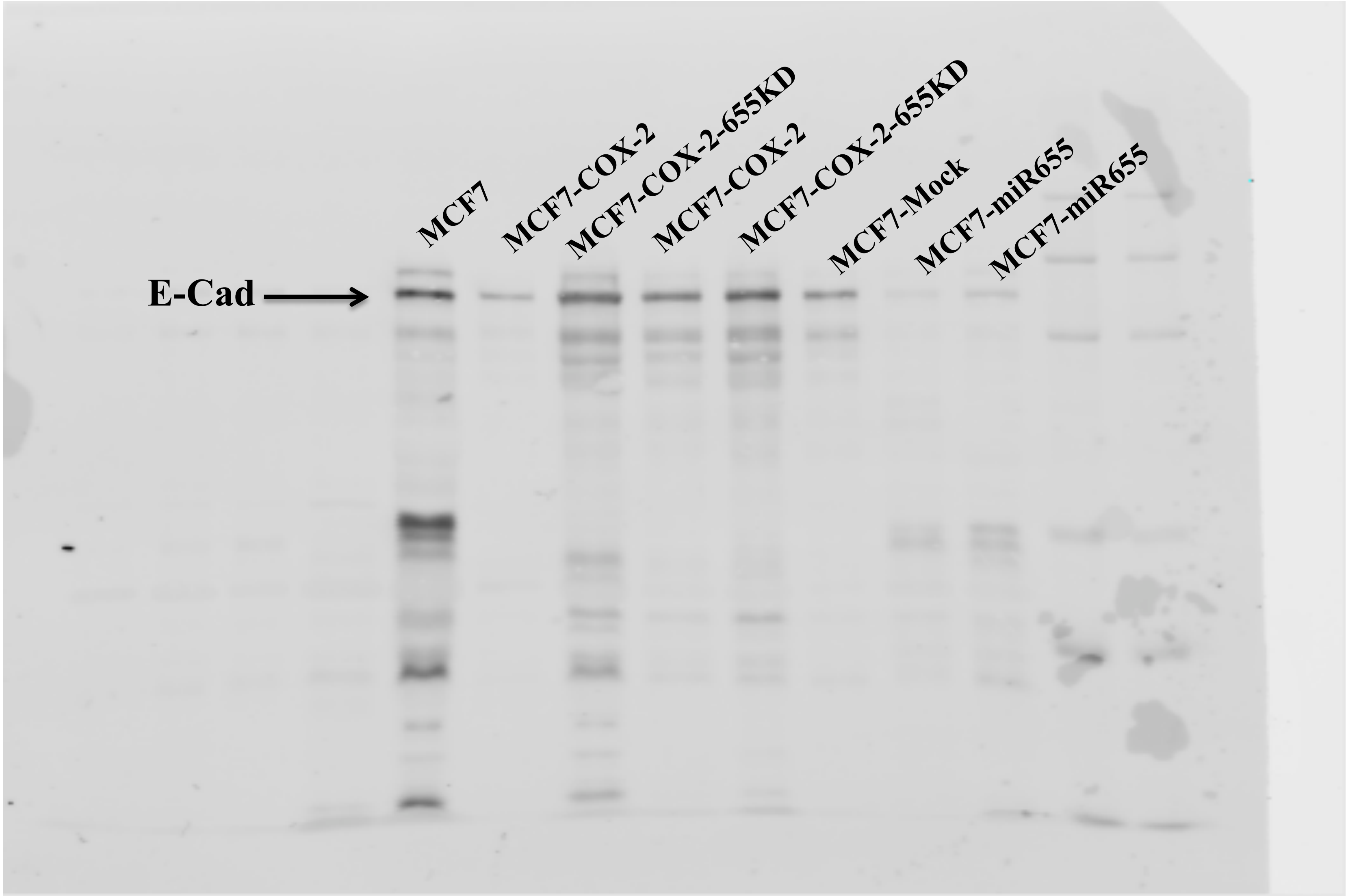

Supplementary Table 1: micro RNA (miRNA) and their target gene list identified with combined gene expression and miRNA expression analysis

Genes downregulated in MCF7-COX2 and targeted by miR655	Fold Change
CPEB (Cytoplasmic polyadenylation element-binding)-2	-1.63889
RILPL2 (Rab interacting lysosomal protein-like 2)	-1.57958
EPB41L2 (erythrocyte membrane protein band 4.1-like 2)	-1.95417
OPTN (optineurin)	-2.22335
TP53INP1 (tumor protein p53 inducible nuclear protein)	-1.67759
IFIT2 (interferon-induced protein with tetratricope)	-5.06359
DNAJB9 (DnaJ (Hsp40) homolog, subfamily B, member 9)	-1.51912
FGD6 (FYVE, RhoGEF and PH domain containing 6)	-2.09673
WIPF1 (WAS/WASL interacting protein family, member)	-3.07156
NDRG1 (N-myc downstream regulated 1 (NDRG1), tra)	-1.62012

List of all genes targeted by miR655: ADAM10, ADAMTS6, ADM, AFF4, AHCYL2, AHDC1, AHRR, AKIRIN2, AMMECR1L, ANK2, ANKRD13C, ANKRD28, ANKS1B, AP1S3, AQP11, ARID4B, ARL3, ARPP-21, ARSB, ASCL1, ATG12, ATMIN, BCL2L2, BCL6, CALM1, CALM2, CANX, CAPRIN2, CAPZA2, CASC4, CASK, CBL, CBR4, CCDC82, CCPG1, CD47, CDC2L6, CDC42, CEBPB, CHD2, CHUK, CLASP2, CLCF1, CNBP, CNR1, CPD, CPEB2, CPEB3, CPNE8, CREBZF, CTDSP2, CUGBP2, DCN, DDIT4, DMD, DNAJB9, DOCK9, DPY19L1, DYRK1A, ECE1, EIF1B, EIF2S2, EIF4A2, EIF4E, EPB41L2, EPB41L5, EPHA4, ERRFI1, ETV1, FAM118B, FAM19A2, FAS, FBXO45, FERMT2, FEZ2, FGA, FGD6, FIGNL2, FNDC3B, FNIP1, FOXO1, FRAS1, FZD4, FZD5, GABPA, GMFB, GNAI3, GOPC, GPATCH8, GPD2, GPR177, GRB10, GRIA4, GRM2, GTF2E1, HBS1L, HDAC9, HECA, HEG1, HES1, HOOK3, HORMAD1, IFIT2, ING2, INHBB, INSIG2, IREB2, JHDM1D, JUN, KCMF1, KCNJ13, KCTD1, KCTD21, KDELR2, KIAA0101, KIAA0355, KIAA0831, KIAA1012, KIAA1468, KLF3, KLHL24, KLHL3, KPNA6, LBA1, LIMK1, LIN54, LMO4, LPHN2, LPHN3, LRP1B, MAFB, MAGI2, MAL2, MAP2K4, MAP4K4, MAPK1, MAPRE1, MARCKS, MDFIC, MEIS1, MEX3B, MID1, MOBKL2B, MON2, MRPS14, MXD4, MYO10, NAB1, NAP1L5, NBR1, NCOA1, NCOA3, NDEL1, NDFIP1, NDRG1, NEDD4, NF1, NFASC, NFYB, NTNG1, ODZ1, OPA1, OPTN, PAG1, PBX3, PCDH7, PCDHAC1, PCTK2, PDGFA, PDIK1L, PELI2, PFTK1, PGAP1, PHF20, PIK3C2A, PIK3R1, PLCXD3, PMEPA1, PNN, PNRC1, PPAP2B, PPP1R12A, PRICKLE2, PRKD1, PTCH1, PTP4A1, PTPN11, PTTG1, PTX3, RAB10, RAB2A, RAB3GAP2, RAB5B, RANBP9, RAP2C, RBM9, RDH10, REEP1, RGS7, RILPL2, RNF111, RNF114, RNF44, RPS6KB1, RSBN1, RTN1, SAMD12, RAS1, SATB2, SC5DL, SCARB2, SEC22B, SEMA3A, SENP5, SERTAD2, SESTD1, SETD7, SFRS5, SFRS7, SHANK2, SHOX2, SIAH1, SKP1, SNAI2, SNAPC1, SOCS2, SOX4, SP8, SPAG7, SRPK2, SSX2IP, STK38L, SYNJ1, SYT16, TBC1D9, TGFBR2, TIMP2, TLE4, TLK2, TMEM65, TNPO1, TOB1, TP53INP1, TRIB2, TRIM24, TSPAN7, UBA6, UBAP1, UBE2D3, UBE2E2, UBE2E3, UBE2W, VEGFA, VKORC1L1, WBP4, WDR20, WDR33, WIPF1, WNK1, WSB1, XIAP, YAF2, YIPF5, YTHDF3, YWHAZ, ZCCHC13, ZCCHC24, ZFAND5, ZFPM2, ZIC1, ZNF217, ZNF423, ZNF521, ZNF532, ZRANB2
